# Guidelines for the use of survivorship care plans: a systematic quality appraisal using the AGREE II instrument

**DOI:** 10.1186/s13012-015-0254-9

**Published:** 2015-05-03

**Authors:** Sarah A Birken, Shellie D Ellis, Jennifer S Walker, Lisa D DiMartino, Devon K Check, Adrian A Gerstel, Deborah K Mayer

**Affiliations:** Department of Health Policy and Management, Gillings School of Global Public Health, The University of North Carolina at Chapel Hill, 1103E McGavran-Greenberg, 135 Dauer Drive, Campus Box 7411, Chapel Hill, NC 27599-7411 USA; Department of Health Policy and Management, University of Kansas School of Medicine, Mail Stop 3044, 3901 Rainbow Boulevard, Kansas City, KS 66160 USA; Health Sciences Library, The University of North Carolina at Chapel Hill, 335 S. Columbia Street, Chapel Hill, NC 27599-7585 USA; School of Nursing, University of North Carolina at Chapel Hill, 2800 Carrington Hall CB# 7460, Chapel Hill, NC 27599 USA

**Keywords:** Survivorship care plan, Guidelines, Dissemination, Quality appraisal

## Abstract

**Background:**

Survivorship care plans (SCPs) are written treatment summaries and follow-up care plans that are intended to facilitate communication and coordination of care among survivors, cancer care providers, and primary care providers. A growing number of guidelines for the use of SCPs exist, yet SCP use in the United States remains limited. Limited use of SCPs may be due to poor quality of these guidelines. The purpose of the study was to evaluate the quality of guidelines for SCP use, tools that are intended to promote evidence-based medicine.

**Methods:**

We conducted a comprehensive search of the literature using MEDLINE/PubMed, EMBASE (Excerpta Medica Database), and CINAHL (Cumulative Index to Nursing and Allied Health Literature) published through April 2014, in addition to grey literature sources and bibliographic and expert reviews. Guideline quality was assessed using the AGREE II instrument (*A*ppraisal of *G*uidelines for *R*esearch and *E*valuation, 2nd edition), a tool developed by an international group of scientists to advance the quality of clinical practice guidelines. To promote consistency with extant studies using the AGREE II instrument and to clearly and unambiguously identify potentially useful guidelines for SCP use, we also summarized AGREE II scores by strongly recommending, recommending, or not recommending the guidelines that we evaluated.

**Results:**

Of 128 documents screened, we included 16 guidelines for evaluation. We did not strongly recommend any of the 16 guidelines that we evaluated; we recommended 5 and we did not recommend 11. Overall, guidelines scored highest on clarity of presentation (i.e., guideline language, structure, and format): Guidelines were generally unambiguous in their recommendations that SCPs should be used. Guidelines scored lowest on applicability (i.e., barriers and facilitators to implementation, implementation strategies, and resource implications of applying the guideline): Few guidelines discussed facilitators and barriers to guideline application; advice and tools for implementing guidelines were vague; and none explicitly discussed resource implications of implementing the guidelines.

**Conclusions:**

Guidelines often advocated survivorship care plan use without justification or suggestions for implementation. Improved guideline quality may promote survivorship care plan use.

**Electronic supplementary material:**

The online version of this article (doi:10.1186/s13012-015-0254-9) contains supplementary material, which is available to authorized users.

## Background

The transition from cancer treatment to follow-up care is often challenging for the nearly 15 million cancer survivors in the United States [1]. To facilitate survivors’ transitions, the Institute of Medicine (IOM) recommends that cancer care providers develop and deliver to survivors and their primary care providers survivorship care plans (SCPs). SCPs are written documents that are often developed in cancer programs and, ideally, include plans for follow-up care, such as surveillance and preventive services, and supporting information such as survivor’s diagnosis, stage, and cancer treatments received. SCPs are intended to facilitate communication and coordination of care among survivors, cancer care providers, and primary care providers [2]. SCP use is increasingly advised and required in guidelines issued by cancer care quality improvement organizations (e.g., Commission on Cancer) [2]. Despite increasingly pervasive guidelines for SCP use, a recent survey indicated that only 20% of United States oncologists reported always/almost always providing SCPs [3]. Further, many providers develop SCPs without delivering them to survivors or their primary care providers [4].

Limited SCPs use in practice may relate, in part, to poor quality of guidelines for SCP use. Guidelines are tools that are intended to promote the use of recommended practices. High-quality guidelines reflect the perspectives of relevant stakeholder groups; provide clear guidance for implementation; are based on empirical evidence, explicit in their methods of development, critically reviewed by experts, and free from conflicts of interest; and are specific and unambiguous [5]. Evidence suggests that providers’ intentions to implement guidelines are stronger when guidelines are clear and unambiguous [6,7]. Clear, unambiguous guidelines for the use of SCPs might define for whom and by whom SCPs should be developed, when and where SCPs should be developed and delivered, to whom SCPs should be delivered, and what survivors and primary care providers should do with SCPs once they receive them. Evidence of limited and inconsistent SCP use may suggest that cancer care providers lack clear guidance regarding these questions [3,8]. Optimal responsibility and timing for developing and delivering SCPs are unclear [3,9]; the utility of electronic SCPs has been debated [3,9-11]; and questions remain regarding where SCPs are optimally delivered (e.g., survivorship clinic, final treatment visit) [12,13].

The purpose of this study was to assess the quality of guidelines for SCP use. Results may offer perspective on why SCP use has been limited to date. If the quality of guidelines for the use of SCPs is low, then developing clearer, less ambiguous guidelines may represent a first step toward promoting SCP use. If the quality of guidelines for the use of SCPs is high, then efforts may need to focus on multifaceted interventions to promote the implementation of guidelines for SCP use in practice.

## Methods

### Literature search

For the purposes of this study, we used the IOM’s definition of guidelines: “statements that include recommendations intended to optimize patient care” ([14], p. 25). For inclusion in this study, we required guidelines to include recommendations regarding the development, delivery, and/or use of SCPs during follow-up care.

A broad range of literature was gathered to identify guidelines for the use of SCPs. The following electronic databases were searched for references to guidelines related to SCPs published through April 15, 2014: MEDLINE/PubMed (1946–2014), EMBASE (Excerpta Medica Database) (1947–2014), and CINAHL (Cumulative Index to Nursing and Allied Health Literature) (1981–2014). In addition to the databases for indexed scientific publications, we searched grey literature sources, including websites of professional organizations and guidelines groups (see Additional file 1). The search used a broad strategy that combined terms for ‘survivorship care plans’ and ‘guidelines’.

Our initial search yielded 175 unduplicated records. An additional 39 references were identified through searches of guidelines groups’ and professional organizations’ websites and publications. Additional file 1 identifies these websites and publications and depicts our process of excluding records that did not contain recommendations regarding the use of SCPs. Eighty-six duplicates were removed, yielding 128 records. From this, we eliminated 111 records that represented guidelines for the use of clinical procedures, non-English publications, childhood cancers, adult survivors of pediatric cancers, models, programs, tools, editorials, dissertations, and templates not accompanied by explicit SCP recommendations. This yielded 17 unique records.

We sent the titles of the 17 records to six experts in the field of cancer survivorship for review. These experts, along with SB and DM, also experts in survivorship, were asked to review the list to ensure accuracy and comprehensiveness. Experts suggested that the authors investigate six additional resources that they believed might reveal additional guidelines; three of these met the inclusion criteria applied in previous rounds of the search, resulting in 20 records that were included in our final full-text review.

SB and SE independently conducted full-text review of the 20 records, using two criteria for inclusion: Records were required to constitute a guideline per the IOM’s definition [14] and to include recommendations regarding the use of SCPs. Disagreements were resolved through consensus and review by DM. Four records were excluded during this process, yielding 16 guidelines to be evaluated.

### Data abstraction

Guidelines evaluated in the study included recommendations regarding topics other than SCP use. Since the purpose of this study was to evaluate the quality of guidelines for SCP use, we did not abstract data regarding recommendations that did not relate to SCP use. To extract data from guidelines related to SCP use, SB developed a data extraction form (see Additional file 2) based on domains specified in the AGREE II instrument (*A*ppraisal of *G*uidelines for *R*esearch and *E*valuation, 2nd edition; www.agreetrust.org; domains described in detail in the analysis section), a tool developed by an international group of scientists to advance the quality of clinical practice guidelines [15]. The form was reviewed and edited by all authors.

SB and LD began by collaboratively extracting data from one guideline. Then, we independently extracted data from a second guideline and met to resolve discrepancies. SB and LD then independently extracted data from the remaining 14 guidelines. SE synthesized SB and LD’s extracted data into a single form.

### Analysis

We assessed the overall quality of the 16 included guidelines using the AGREE II instrument. The validity and reliability for assessment of practice guidelines using the AGREE II have been established [15,16]. The instrument includes 23 items that address six quality domains: (1) scope and purpose, (2) stakeholder involvement, (3) rigor of development, (4) clarity of presentation, (5) applicability, and (6) editorial independence. Two additional assessment items (Overall Guideline Assessment) pertain to an overall judgment of the guideline. Each item is rated on a seven-point Likert scale, with 1 assigned for items with no clear discussion and 7 for exceptional quality of reporting. DC and LD read the entire AGREE II user’s manual and then independently rated all included guidelines.

Not all of the AGREE II items were applicable to guidelines for SCP use. Items 11 (“The health benefits, side effects, and risks have been considered in formulating the recommendations.”) and 16 (“The different options for management of the condition or health issue are clearly presented.”) are limited in their applicability to SCPs; evidence of benefits, risks, and alternatives is limited. As such, these items were excluded from analysis. DC, LD, and SB worked together to revise item 12 (“There is an explicit link between the recommendations and the supporting evidence.”); in light of limited empirical evidence regarding SCPs, DC and LD interpreted item 12 as “Evidence is described, and recommendations follow from it”.

SB conducted the final scoring, according to the instrument protocol, by adding together DC and LD’s respective ratings for items in each domain and standardizing the total score out of 100%.

The AGREE II manual does not provide guidance regarding how to interpret scores. To promote consistency with extant studies that have used the AGREE II instrument [17,18], we adopted their method: Guidelines receiving a standardized score of 50% or greater on all domains were *strongly recommended*, guidelines receiving an overall assessment of 50% or greater were *recommended*. and guidelines that neither received a standardized score of 50% or greater on all domains nor received an overall assessment of 50% or greater were *not recommended*.

## Results

### Guideline characteristics

Table 1 describes guideline characteristics. Sixteen guidelines were included in the review. Eleven were developed in the United States; the remaining five guidelines were developed in Canada [19], the Netherlands [20], the United Kingdom, [21,22] and Australia [23]. Only three guidelines focused specifically on SCP use [24-26]. In the remaining 13, guidelines for SCP use were tangential; 11 [19-23,27-32] primarily focused on the care of cancer survivors, and two focused on standards for cancer care programs [2,33].

**Table 1:** **Guideline characteristics**

**Guideline**	**Country of origin**	**Focus**	**Target audience**	**Survivor group**
Alberta Health Services	Canada	Survivorship care	Oncology providers; family physicians	Breast
American Cancer Society	United States	Survivorship care	None explicitly stated, but oncology practitioners implied	None specified
American Society of Clinical Oncology	United States	Survivorship care	None explicitly stated, but oncology practitioners implied	None specified
Association of Community Cancer Centers	United States	Guidance for cancer program development	Association of Community Cancer Centers members; cancer care providers; community cancer centers	None specified
Commission on Cancer	United States	Standards for cancer programs	None explicitly stated, but Commission on Cancer members, cancer care providers, and accredited cancer programs implied	None specified
Comprehensive Cancer Center of the Netherlands	Netherlands	Survivorship care	Cancer care professionals who discuss aftercare and the cancer survivorship care plan with patients treated with curative intent	Solid tumor
International Myeloma Foundation	United States	Survivorship care plans	Members of the Nurse Leadership Board; care providers for multiple myeloma survivors; healthcare providers; nurses	Multiple myeloma
Institute of Medicine	United States	Survivorship care	Cancer care providers	Breast, prostate, and colorectal cancers; Hodgkin’s disease
LIVESTRONG	United States	Survivorship care	Practitioners in any effective cancer survivorship program	None specified
National Breast and Ovarian Cancer Centre	Australia	Survivorship care	Health care professionals	Breast
National Comprehensive Cancer Network	United States	Survivorship care plans	Health care professionals who care for survivors of adult onset cancer in the post-treatment period, including those in both oncology and primary care practices	None specified
National Coalition for Cancer Survivorship/Institute of Medicine/ Lance Armstrong Foundation/ National Cancer Institute	United States	Survivorship care plans	Clinical oncology providers; primary care providers	None specified
National Cancer Institute Community Cancer Centers Program	United States	Survivorship care plans	Practitioners in National Cancer Institute Community Cancer Centers Program member programs	None specified
National Collaborating Centre for Cancer	United Kingdom	Survivorship care	Advanced practice nurses; nurses; pharmacists; physician assistants; physicians	Breast
President’s Cancer Panel	United States	Survivorship care	Health care providers	None specified
National Health Service	United Kingdom	Survivorship care	Primary Care Trusts CEs, National Health Service Trust CEs, Strategic Health Authorities CEs, Care Trust CEs, Foundation Trust CEs, Medical Directors, Directors of Nursing, Local Authority CEs, Directors of Adult Social Services, Primary Care Trusts Chairs, National Health Service Trust Board Chairs, Directors of Human Resources, Directors of Finance, Allied Health Professionals, General Practitioners, Communications Leads, Directors of Children’s Social Services (p 2); service users, carers, clinicians and service commissioners. (p 5);	None specified

*CE* chief executives.

### AGREE II appraisal

Table 2 contains scores in each domain based on the AGREE II appraisal. The quality of guidelines for SCP use varied widely but was relatively low overall. We did not strongly recommend any of the 16 guidelines, as they did not receive a standardized score of 50% or greater on all domains. We recommended five, as they received an overall assessment of 50% or greater [19,20,22,25,26]. We did not recommend the remaining 11 guidelines, as they neither received a standardized score of 50% or greater on all domains nor received an overall assessment of 50% or greater.

**Table 2:** **Guideline appraisal**

**Guideline**	**Domain #1: scope and purpose**	**Domain #2: stakeholder involvement**	**Domain #3: rigor of development**	**Domain #4: clarity of presentation**	**Domain #5: applicability**	**Domain #6: editorial independence**	**Overall assessment**	**Recommendation**
Alberta Health Services	61%	35%	83%	63%	0%	75%	58%	Recommended
American Cancer Society	33%	6%	7%	46%	4%	8%	8%	Not recommended
American Society of Clinical Oncology	44%	36%	15%	75%	23%	46%	42%	Not recommended
Association of Community Cancer Centers	19%	36%	2%	75%	4%	0%	17%	Not recommended
Commission on Cancer	19%	33%	12%	88%	19%	0%	33%	Not recommended
Comprehensive Cancer Center of the Netherlands	69%	75%	56%	96%	38%	42%	67%	Recommended
International Myeloma Foundation	56%	42%	17%	63%	21%	96%	42%	Not recommended
Institute of Medicine	22%	42%	12%	92%	38%	38%	50%	Not recommended
LIVESTRONG	33%	22%	10%	96%	31%	0%	33%	Not recommended
National Breast and Ovarian Cancer Centre	28%	58%	26%	46%	0%	63%	33%	Not recommended
National Comprehensive Cancer Network	72%	58%	26%	67%	31%	42%	50%	Not recommended
National Coalition for Cancer Survivorship/Institute of Medicine/Lance Armstrong Foundation/National Cancer Institute	92%	61%	18%	63%	52%	0%	58%	Recommended
National Cancer Institute Community Cancer Centers Program	56%	42%	17%	88%	40%	29%	58%	Recommended
National Collaborating Centre for Cancer	19%	86%	69%	79%	40%	29%	58%	Recommended
President’s Cancer Panel	11%	92%	7%	92%	15%	0%	25%	Not recommended
National Health Service	31%	39%	5%	42%	0%	0%	25%	Not recommended

#### Domain 1: scope and purpose

Scope and purpose criteria relate to the objectives of the guideline, health questions to be addressed, and target population(s) [15]. The average guideline score for this domain was low (42%). Descriptions of objectives were vague (e.g., to provide the most appropriate follow-up care to survivors), and only half of the guidelines described the purpose of an SCP [20,24-30]. The National Coalition for Cancer Survivorship (NCCS)/IOM/Lance Armstrong Foundation/National Cancer Institute (NCI) guideline, which performed best in this domain, described an SCP’s purpose as follows: “[The SCP] summarizes and communicates what transpired during cancer treatment”, functions “to promote a healthy lifestyle to prevent recurrence and reduce the risk of other comorbid conditions”, and “gives patients an opportunity to take some responsibility for their care and may help to ensure adherence to follow-up recommendations” [25]. Only three guidelines described SCP-related health questions specific to SCPs [21,25,30].The NCCS/IOM/Lance Armstrong Foundation/NCI guideline described a number of SCP-specific health questions, including the following: Who is responsible for creating the plan and discussing the plan with patients? What are the respective roles of oncologists, primary care physicians, and nurses? What economic strategies could encourage implementation of care planning? What barriers exist to creating the care plan? How can they be overcome? [25]. Only three guidelines specified a target survivor group [2,27,31].

#### Domain 2: stakeholder involvement

Stakeholder involvement criteria relate to whether guidelines were developed with input from appropriate stakeholders and reflect the views of intended users [15]. On average, guidelines received a score of 48% for this domain. Most guidelines did not adequately describe their guideline development team. Four guidelines did not identify members of the guideline development team [19,21,25,28]. Five guidelines identified the development team but did not identify members’ professional groups [2,25,27,30,33]. Most guidelines did not describe including stakeholders in guideline development, and those that did were vague about stakeholders’ roles. Half of the guidelines recommended particular providers to be primarily responsible for developing and delivering SCPs, but recommendations were inconsistent across guidelines. Four guidelines indicated that oncology providers have primary responsibility for initiating them [2,25,31,32]. In some cases, “oncology providers” referred to the primary oncologist [2,32]; in other cases, “oncology providers” referred to the oncologist responsible for the last course of treatment [32], oncology nurses [31,32], or some combination of physicians and nursing staff [25].

#### Domain 3: rigor of development

Rigor of development criteria relate to methods of gathering and synthesizing evidence considered during guideline development, processes of guideline development, and methods for updating the guideline [15]. Only three guidelines described using systematic methods to search for and review evidence [19,22,23]. Another three guidelines discussed the current lack of definitive evidence on SCP effectiveness [25,30,32]. Two guidelines [27,30] described an explicit link between their recommendations and specific evidence, including studies on SCPs’ effectiveness, cost efficiency, and time- and resource-intensiveness. Most guidelines described guideline development processes, but they did so with varying levels of detail. One guideline, for example, detailed their recommendation development process, describing an expert meeting, the development of a list of essential elements of survivorship care, and consensus-building activities [29]. Others used more general terms (e.g., “Priority topic areas … are determined in consultation with key stakeholders… A specific multidisciplinary Working Group, including consumers, is established for each topic identified and is involved in all aspects of guideline development” [23]). Just half of the guidelines discussed external expert review, and only two [19,20] explicitly discussed procedures for updating their document.

#### Domain 4: clarity of presentation

Clarity of presentation criteria include the following: the recommendations are specific and unambiguous and key recommendations are easily identifiable [15]. On average, guidelines received the highest scores in this domain (73%). All 16 guidelines clearly endorsed recommendations for SCP use; however, they varied in degrees of specificity and lack of ambiguity. One guideline was particularly specific and unambiguous in stating that, in light of lack of evidence of SCPs’ effectiveness, SCP use was recommended but not required [30]. Acknowledgments of such uncertainty are consistent with AGREE II guidance for evaluating Domain 4: “Evidence is not always clear cut and there may be uncertainty about the best care option(s); in this case, the uncertainty should be stated in the guideline”. Most guidelines’ key recommendations were generally readily identifiable (e.g., grouped together in one section, summarized in a box, in boldface, or otherwise emphasized).

#### Domain 5: applicability

Applicability criteria relate to facilitators and barriers to implementing the guideline’s recommendations, strategies for implementing the guideline’s recommendations, and resource implications of implementation [15]. Of all AGREE II domains, guidelines for SCP use received the lowest scores in this domain (22%). Only six guidelines discussed facilitators and barriers to SCP implementation [20,25-27,29,32]. Half of the guidelines [2,20,25-27,29-31] recommended a specific SCP template. All but two of the guidelines [23,28] specified that SCPs should include both a treatment summary and evidence-based follow-up care plan. Most guidelines offered guidance about the timing of SCP development and delivery. Of these, nine guidelines [2,20-22,25,26,30-32] specified that SCPs should be delivered upon completion of active treatment and the transition to the post-treatment period; however, definitions of this period varied from upon completion of primary cancer treatment and treatment for recurrence [31,32] to first course of cancer treatment [2] to completion of active treatment and subsequent transition points (e.g., further disease; the move toward end-of-life care) [21]. Although resource-intensiveness was identified as a potential barrier to successful SCP implementation, resource implications were not explicitly discussed in any of the guidelines. Only one guideline offered auditing criteria for measuring adherence to SCP recommendations (e.g., the percentage of survivors in a practice with a SCP) [22].

#### Domain 6: editorial independence

Editorial independence criteria relate to ensuring lack of bias in the development of the guideline [15]. The average score for editorial independence was low (29%). Most guidelines did not provide a statement regarding funding, its influence on recommendations, or conflicts of interest. Of guidelines that included a funding acknowledgment, only two explicitly stated that their funding source did not influence the recommendations [30,33].

#### Overall assessment

Overall guideline quality was low. Scores ranged from 8% [28] to 67% [20]. Only five guidelines [19-21,25,26] received an overall score above 50%. Thus, we only recommended these five guidelines and strongly recommended none.

## Discussion

Overall, AGREE II domain scores suggest that the quality of guidelines for SCP use is low. This finding is consistent with that of other studies that have shown poor quality of guidelines in cancer care [34]. Guidelines were generally definitive in their recommendations to use SCPs, but these recommendations were often not explicitly linked to evidence. Further, guidelines for SCP use offered little clarity regarding why, when, where, and how SCPs should be used; who should use them; and for whom they should be used. Only half of the guidelines explained the purpose of SCPs [20,24-30]. Recommendations regarding when, where, and by whom SCPs should be used varied across guidelines. Most guidelines recommended that SCPs be developed and delivered upon survivors’ transition to follow-up care, but they differed in their definitions of the transition period; only four guidelines indicated who should develop and deliver SCPs [2,25,31,32]; and just six specifically identified the cancer survivors to whom their guideline applied [19,20,22-24,32].

The lack of clarity was underscored by the challenges of abstracting guideline data. Two investigators abstracted data, and a third reconciled abstractions. In multiple instances, the independent abstractions led to two reasonable but distinct interpretations of the guidelines. That two informed individuals could develop such distinct interpretations of one guideline suggests that busy clinicians may have difficulty culling relevant information that is consistent with their peers’ practices. This lack of guideline clarity may partly explain limited and inconsistent SCP use in practice [4,35].

As we suggested might be the case in the introduction, the poor quality of guidelines for SCP use may contribute to limited and inconsistent SCP use in practice [3,8]. Clear and unambiguous guidelines for SCP use may promote the effectiveness with which SCPs are implemented [6,7]; in effect, high-quality guidelines are an implementation strategy [36]. In particular, guidelines that use behaviorally specific terms may be the most effective way of increasing implementation [6]. Guidelines for the use of SCPs may be improved with precise and consistent definitions of which templates are best to use; to whom the guideline applies; to whom the guideline does not apply; and when, where, and by whom SCPs may be most effectively developed and delivered. Clear specifications may facilitate evaluation of adherence to guidelines. Future research should assess the relationship between guideline quality and the effectiveness of SCP implementation.

Many of the guidelines included in this study scored highly in some, but not all, domains. Efforts to promote SCP use may benefit from combining elements from multiple guidelines into a single clear, unambiguous resource. SB, JW, LD, and colleagues are currently synthesizing information from the guidelines that scored highest in each AGREE II domain in this study to create a “meta-guideline” for SCP use that practitioners may reference to facilitate their decision regarding adopting SCPs and, should they adopt SCPs, to facilitate their implementation. By leveraging the work of existing guidelines, this effort represents an efficient approach to facilitating practitioners’ decisions around SCP adoption and implementation.

Study limitations should be considered when interpreting results. The search was conducted by a librarian with expertise in systematic reviews; however, the search may have excluded some guidelines. For example, we excluded guidelines that were not published in English. And although the AGREE II instrument is a useful tool for evaluating guideline quality, it has limitations. In particular, it does not offer guidance for interpreting scores. Our criteria for strongly recommending, recommending, or not recommending guidelines were intended to promote consistency with extant studies using the AGREE II instrument [17,18] and to clearly and unambiguously identify potentially useful guidelines for the use of SCPs. In most cases, this resulted in overall assessment scores that were commensurate with scores received on individual domains. For this reason, the field of guideline assessment may benefit from future AGREE II studies using a similar method. Further, AGREE II only assesses the quality of guidelines’ structure and content; it does not assess the quality of guidelines’ recommendations. A case in point, despite the variation in guidelines’ structural and content quality that we found in this study, we found almost no variation in guidelines’ recommendations in favor of SCP use. As such, although our assessment of the guidelines indicates room for improvement to facilitate implementation of recommendations, neither our study nor the AGREE II instrument offers insight into whether the included guidelines’ recommendations are correct.

Another challenge of AGREE II relates to applying criteria across several guidelines. Scores are intended to be based on a guideline’s quality irrespective of other guidelines’ quality. However, as scoring proceeds, reviewers’ evaluations of guidelines becomes biased by familiarity with previously scored guidelines. The quality of future AGREE II evaluations may benefit from randomizing the order in which each reviewer evaluates guidelines to average out the effects of increased familiarity.

## Conclusions

Our findings indicate that the quality of guidelines for SCP use is poor. Guideline quality may be improved with more behaviorally specific information regarding methods for effective SCP implementation. Organizations that develop cancer-related guidelines may promote quality by incorporating AGREE II into their formal practice guideline programs, as many other guideline-developing organizations have successfully done [15].

## Additional files

ᅟ Additional file 1:
**Literature review flow diagram.** Diagram of literature sources and review process. Additional file 2:
**Data extraction form.** Form used to extract data from guidelines related to SCP use.

## Abbreviations

ACS: American Cancer Society
ACCC: Association of Community Cancer Centers
ASCO: American Society of Clinical Oncology
CoC: Commission on Cancer
IOM: Institute of Medicine
NBOCC: National Breast and Ovarian Cancer Centre
NCCN: National Comprehensive Cancer Network
NCCS: National Coalition for Cancer Survivorship
NCI: National Cancer Institute
NCCCP: NCI Community Cancer Centers Program
NHS: National Health Service
