# Supplementary material for: Guidelines for the use of survivorship care plans: a systematic quality appraisal using the AGREE II instrument
Source: Implement Sci. 2015 May 3;10:63. doi: 10.1186/s13012-015-0254-9 (PMC4425878; doi:10.1186/s13012-015-0254-9)
Supplement: Additional file 1: — Literature review flow diagram. Diagram of literature sources and review process. [file 13012_2015_254_MOESM1_ESM.docx]

**Additional file 1.** Literature review flow diagram

Excluded based on criteria: n = 111

Clinical practice guidelines, non-English publications, childhood cancers, models, programs, tools, editorials, dissertations

Records screened through title/abstract review

n = 128

*Guideline groups:

Guidelines/reports/recommendations included in final analysis

n = 16

Excluded based on criterion: n = 4

Does not specifically recommend survivorship care plan use

Guidelines/reports/recommendations evaluated in final full text review

n = 20

Additional guidelines/reports/recommendations identified by subject matter experts‡

n = 3

Guidelines/reports/recommendations identified from literature search

n = 17

Published Literature:

PubMed/MEDLINE: n = 55

EMBASE: n = 98

CINAHL: n = 22

Total: n = 175

Grey Literature:

Guideline groups*: n = 6

Professional organizations†: n = 32

Total: n = 39

Duplicates removed

n = 86

- Agency for Healthcare Research and Quality’s National Guideline Clearinghouse: n = 6 (Clinical guidelines; excluded)
- National Institute for Health and Care Excellence: n = 0
- New Zealand Guidelines Group: n = 0
- Scottish Intercollegiate Guidelines Network: n = 0

†Professional organizations:

- American Cancer Society and George Washington University Cancer Institute: National Cancer Survivorship Resource Center: n = 1 (<http://www.cancer.org/treatment/survivorshipduringandaftertreatment/nationalcancersurvivorshipresourcecenter/index>)
- BC Cancer Agency: n = 1

[Cancer Survivorship: creating uniform and comprehensive supportive care programming in Canada- Cancer Transitions: Moving Beyond Treatment](http://www.bccancer.bc.ca/NR/rdonlyres/E6F649B9-761C-4C51-89E0-C2F0834B8DCC/46387/FINALCPACReport2010CancerTransitions_July192010_.pdf).

- Cancer Australia: n = 0
- Cancer Care Ontario = 1 (<https://www.cancercare.on.ca/cms/one.aspx?objectId=124833&contextId=1377>)
- Canadian Cancer Society, Nationwide Strategic Plan: n = 1 <http://www.cancer.ca/~/media/cancer.ca/CW/about%20us/nationwide%20strategic%20plan/CCS%20Nationwide%20Strategic%20Plan%20EN.pdf>
- Canadian Partnership Against Cancer: n = 1

[Survivorship Empowerment Model for Integrated Cancer Care](http://www.cancerview.ca/cv/portal/Home/QualityAndPlanning/QPProfessionals/HealthHumanResources/ServiceDeliveryModelsDatabase/SDMDDetail?contentid=10337) (a model, not a guideline)

- Department of Health and Human Services/Centers for Disease Control and Prevention/Lance Armstrong Foundation. n = 1

A National Action Plan for Cancer Survivorship: Advancing Public Health Strategies. ([www.cdc.gov/cancer/survivorship/pdf/plan.pdf](http://www.cdc.gov/cancer/survivorship/pdf/plan.pdf))

- Department of Health, Macmillan Cancer Support and NHS Improvement (2010) The National Cancer Survivorship Initiative: n = 1 <http://webarchive.nationalarchives.gov.uk/20130107105354/http://www.dh.gov.uk/prod_consum_dh/groups/dh_digitalassets/@dh/@en/@ps/documents/digitalasset/dh_111477.pdf> (principles for improved care but not necessarily guidelines)
- European Partnership for Action Against Cancer: n = 22

(http://www.epaac.eu/national-cancer-plans)

- European Society for Medical Oncology: n = 0
- International Myeloma Foundation Nurse Leadership Board: n = 1 <http://ons.metapress.com/content/5417q738x71j8063/fulltext.pdf>
- Improving Outcomes: a strategy for cancer: n = 1

<https://www.gov.uk/government/uploads/system/uploads/attachment_data/file/213785/dh_123394.pdf>

- Journey Forward: n = 0

[Provides free, online tools](http://www.journeyforward.org/about-journey-forward) for building/creating SCP’s, and can be utilized by both health care professionals and patients.  They partner with NCCS, Oncology Nursing Society and several others. They do not have their own independent guidelines.

- MD Anderson: n = 2

[Survivorship: Nutrition Guidelines for Cancer Survivors](http://www.mdanderson.org/patient-and-cancer-information/cancer-information/cancer-topics/survivorship/survivorship-nutrition-guide.pdf) [cancer survivorship algorithms](http://www.mdanderson.org/education-and-research/resources-for-professionals/clinical-tools-and-resources/practice-algorithms/survivorship-algorithms.html)

- National Health and Medical Research Council: n = 0
- University of Pennsylvania: n = 0

Part of the LIVEstrong network, they, too, provide a plan building tool like Journey Forward.  Their use of SCPs is in concordance with guidelines provided by the Institute of Medicine, Children’s Oncology Group, National Cancer Institute, and American Society of Clinical Oncology.

‡Subject Matter Experts (contacted and responded):

1. Sarah Birken
2. Deborah Mayer
3. Patricia Ganz
4. Eva Grunfeld
5. Michael Jefford
6. Mary McCabe
7. Daniel McKellar
8. Julia Rowland
